# Effect of vitamin D supplementation on COVID-19 patients: A systematic review and meta-analysis

**DOI:** 10.3389/fnut.2023.1131103

**Published:** 2023-03-07

**Authors:** Ying Zhang, Jing Li, Min Yang, Qin Wang

**Affiliations:** Department of Endocrinology, West China Hospital of Sichuan University, Chengdu, Sichuan, China

**Keywords:** vitamin D, meta-analysis, COVID-19, mortality, ICU admission, mechanical ventilation, intubation

## Abstract

**Objective:**

To systematically evaluate the impact of vitamin D supplementation on mortality, ICU admission, and the rates of mechanical ventilation or intubation among COVID-19 patients.

**Data sources and study selection:**

The PubMed, Embase, Cochrane Library, CBM, CNKI, VIP, and WanFang databases were searched from 1 December 2019 to 31 December 2022. The authors sought to identify randomized controlled trials and cohort studies that examined the relationship between vitamin D supplementation and mortality, ICU admission, and mechanical ventilation or intubation rates among COVID-19 patients.

**Data extraction and synthesis:**

Two investigators independently searched the literature, extracted the data, and assessed the quality of the included studies. The Grading of Recommendation, Assessment, Development, and Evaluation approach was used to evaluate the quality of the evidence. Meta-analysis was conducted using RevMan 5.3, STATA 15.1, and R 4.1.3 software.

**Results:**

Eight randomized controlled trials (RCTs) and eight cohort studies were included, involving 3359 COVID-19 patients. The pooled analysis of randomized controlled trials showed that vitamin D supplementation did not have a significant effect on reducing mortality (Relative Risk, RR = 0.94, 95% CI 0.69–1.29, *P* = 0.7), while the results of cohort studies indicated that vitamin D supplementation had a positive impact on reducing mortality among COVID-19 patients (RR = 0.33, 95% CI 0.23–0.47, *P* < 0.001). There was no statistically significant difference in the rates of ICU admission (RCTs: RR = 0.64, 95%CI 0.38–1.08, *P* = 0.10; cohort studies: RR = 0.32, 95% CI 0.08–1.29, *P* = 0.109) or rates of mechanical ventilation or intubation (RCTs: RR = 0.77, 95% CI 0.58–1.02, *P* = 0.07; cohort studies: RR = 0.93, 95% CI 0.55–1.58, *P* = 0.789).

**Conclusion:**

The results of this systematic review and meta-analysis suggest that vitamin D supplementation does not have a significant impact on reducing mortality, ICU admission, and the rates of mechanical ventilation or intubation among COVID-19 patients. However, due to the limited number and quality of the studies included, further high-quality studies are needed to confirm these findings.

**Systematic review registration:**

www.crd.york.ac.uk, identifier CRD42021299521.

## Introduction

The global outbreak of coronavirus disease 2019 (COVID-19) has caused a major health crisis with 655,689,115 confirmed cases and 6,671,624 confirmed deaths as of 3 January 2023 (1). The infection caused by severe acute respiratory syndrome coronavirus 2 (SARS-CoV-2) leads to a wide range of symptoms, and patients with comorbidities such as diabetes, cardiovascular disease, and hypertension may face adverse outcomes (2), including ICU admission, mechanical ventilation or intubation, and death.

While vaccines and antiviral drugs have demonstrated efficacy against COVID-19 (3), additional measures, such as vitamin D supplementation, continue to play an important role in managing the disease. Low serum 25-hydroxycholecalciferol [25(OH)D] levels have been linked to increased susceptibility to novel coronavirus infection and greater severity of COVID-19 symptoms (4). Some studies have suggested that vitamin D supplementation may reduce mortality in COVID-19 patients (5, 6), but a previous meta-analyze published in the year 2022 has failed to reach a definitive conclusion due to limited studies and inconsistent study design (7).

With the ongoing spread of COVID-19, the number of clinical studies on the effect of vitamin D supplementation on COVID-19 outcomes has increased (5, 6, 8–13) but the results remain conflicting. Thus, it is necessary to conduct an updated meta-analysis of randomized controlled trials and cohort studies to determine the impact of vitamin D supplementation on mortality, ICU admission, and mechanical ventilation or intubation rates in COVID-19 patients.

## Materials and methods

The present meta-analysis was conducted following the PRISMA (Preferred Reporting Items for Systematic Reviews and Meta-Analysis) statement (14) and has been registered on the international database of prospectively registered systematic reviews, PROSPERO (Registration number: CRD42021299521).

### Inclusion and exclusion criteria

Population: COVID-19 patients of all ages and severity levels.

Intervention: Vitamin D supplements of various forms, analogs, doses, and follow-up durations after the diagnosis of COVID-19.

Comparison: Without vitamin D supplements.

Outcomes: mortality, ICU admission rates, and rates of mechanical ventilation or intubation of COVID-19 patients.

Study design: Randomized controlled trials and cohort studies.

Exclusion criteria: (1) Repeated publications; (2) missing outcome data in the literature; (3) lack of definite Vitamin D dose in each study; and (4) the data are wrong or cannot be extracted.

### Search strategy

The literature search was conducted across multiple databases including PubMed, Cochrane Library, Embase, CNKI, CBM, WanFang Data, and Cqvip, covering the period from 1 December 2019 to 31 December 2022. Search keywords: Dihydroxyvitamin D, Dihydroxyvitamin, Calcitriol, Alfacalcidol, 24,25-Dihydroxyvitamin D, paricalcitol, Dihydroxycholecalciferol, 1 alpha,25-Dihydroxyvitamin, 1alpha,25-Dihydroxycholecalciferol, 1,25-Dihydroxyvitamin, 25Hydroxyvitamin D3, 1, 25-dihydroxy vitamin D, 25-Hydroxyvitamin D3, 25-hydroxyvitamin D, Calcidiol, Calcifediol, Hydroxycholecalciferol, Ergocalciferol, Cholecalciferol, Vitamin D3, Vitamin D2; COVID-19, COVID19, COVID-19 Virus, COVID-19 Virus Disease, COVID-19 Virus Infection, 2019-nCoV Infection, Coronavirus Disease-19, Coronavirus Disease 19, 2019 Novel Coronavirus Disease, 2019 Novel Coronavirus Infection, 2019-nCoV Disease, Disease 2019, Coronavirus, SARS Coronavirus 2 Infection, SARS-CoV-2 Infection, COVID-19 Pandemic. The search terms are described in the Supplementary Text 1.

### Study selection and data extraction

Two investigators independently searched the literature, extracted the data, cross-checked the data, and consulted a third party to resolve any disagreements. The titles and abstracts of the literature were initially screened, followed by a full-text review to determine final inclusion based on the established inclusion and exclusion criteria. The extracted data included (1) the first author, year of publication, location, and date of the study; (2) baseline characteristics and interventions of subjects; and (3)outcome indicators and data, including mortality, ICU admission rates, and mechanical ventilation or intubation rats in COVID-19 patients.

### Risk of bias assessment

The assessment of the risk of bias in the included literature was carried out independently by two investigators, and the results were verified through cross-checked. The risk of bias in cohort studies was evaluated using the Robin-I tool by the Cochrane guidelines for non-randomized studies (15), and RCTs were evaluated by the Cochrane Collaborations Tool For Assessing Risk of Bias recommended by the Cochrane Manual 5.1.0 (16).

### Statistical analysis

RevMan (version 5.3) software (Cochrane Collaboration, UK), Stata (version 15.1) software (Stata Corporation, Lakeway, TX, USA) and R software (version 4.1.3) were used for meta-analysis. The effect size was analyzed using relative risk (RR) and a 95% confidence interval (CI). Hazard ratio (HR) was considered as RR in the study, and the following formula was used to convert odds ratio (OR) into RR: RR = OR/[(1 − Po) + (Po × OR)], where Po represents the incidence of the outcome of interest in the non-exposed group (17). The standard error of the resulting converted RR was calculated using the formula: SElog(RR) = SElog(OR) × log(RR)/log(OR). The adjusted HR or RR and 95% CI were utilized to reduce the impact of confounding factors if available. Otherwise, unadjusted HR or RR was adopted.

The heterogeneity of the included studies was analyzed using the Q test, and if *I*^2^ < 50% and *P* > 0.1, all studies were considered homogenous and the data were analyzed by a fixed-effect model. In case of *I*^2^ ≥ 50% and *P* ≤ 0.1, indicating the presence of heterogeneity, data were analyzed using a random effects model. Potential publication bias was evaluated through funnel plots and Egger’s test.

Stratified analyses were performed based on the type of study design, and sensitivity analyses were conducted to test the reliability of the combined analysis of adjusted/unadjusted RR.

### Quality of evidence

The quality of the evidence was evaluated using the Grading of Recommendation, Assessment, Development, and Evaluation (GRADE) approach (18, 19) and was classified as *high*, *moderate*, *low*, or *very low* based on the following domains: study design, risk of bias, inconsistency, indirectness, imprecision, and other considerations (such as evidence of publication bias). The results are presented in Table 2.

**TABLE 1:** The characteristics of eligible studies.

Study and Country	Type of study and patients source	Intervention and Control	Vitamin D supplements		Control		Number of deaths/Intubation or Mechanical ventilation requirement/ICU admission: number of intervention or control
			Age	25(OH)D levels before/after treatment(ng/ml)	Age	25(OH)D levels before/after treatment(ng/ml)	
Elamir et al. (8), Israel	RCT, Hospitalized patients	Oral 0.5 ug calcitriol per day. vs. Without vitamin D supplements	69 ± 18	NA	64 ± 16	NA	0/0/5: 25 vs. 3/2/8: 25
Cannata-Andía et al. (9), Multicentre	RCT, Hospitalized patients	A single oral dose of 100,000 IU cholecalciferol vs. Without vitamin D supplements	59.0(49.0, 70.0)	17.0(11.8,22.0)/29.0 (20.3,35.0)	57.0(45.0, 67.0)	16.1(11.5, 22.0)/16.4(11.8, 23.0)	22/NA/47: 274 vs. 15/NA/44: 269
Javier Mariani et al. (10), Argentina	RCT, Hospitalized patients	A single oral dose of 500,000 IU of vitamin D3 vs. Placebo	59.8 ± 10.7	32.5 (27.2–44.2)/102 (85.2 to 132.2)^a^	58.3 ± 10.6	30.5(22.5–36.2)/30.0 (27.5–31.0)^a^	5/5/9: 115 vs. 2/6/11: 103
IMurai et al. (20), Brazil	RCT, Hospitalized patients	A single oral dose of 200,000 IU cholecalciferol vs. Placebo	56.5 ± 13.8	21.2 ± 10.1/44.4 ± 15.0	56 ± 15	20.6 ± 8.1/19.8 ± 10.5	9/9/19: 119 vs. 6/17/25: 118
Jessie Zurita-Cruz et al. (21), Mexico	RCT, Hospitalized patients	1,000 IU/day of Cholecalciferol for children younger than 1 year and 2,000 IU/day for 1–17 years. vs. Without vitamin D supplements	10.66(4.41–14.62)	13.8(10.75–18.35)/NA	13.95(7.35-14.87)	11.4(8.7–13.1)/NA	1/NA/NA:20 vs. 6/NA/NA:25
Mikhail V. Bychinin et al. (22), Russia	RCT, Hospitalized patients with hypovitaminosis D	60,000 IU cholecalciferol once per 7 days, followed by daily doses of 5,000 IU vs. Placebo	64.5 (57–71)	9.6(5.6–21)/20.6 (11.8–24.8)	63.5 (54–81)	11.2(8.6–14.9)/10.4 (5.8–12.2)	19/33/NA: 52 vs. 27/37/NA: 54
Castillo et al. (23), Spain	RCT, Hospitalized patients	Oral 0.532 mg Calcifediol on day 1, 0.266 mg on days 3 and 7, then weekly. vs. Without vitamin D supplements.	53.14 ± 10.77	NA	52.77 ± 9.35	NA	0/NA/1:50 vs. 2/NA/13: 26
Sophie De Niet et al. (24), Belgium	RCT, Hospitalized patients with hypovitaminosis D	Oral 25,000 IU of Cholecalciferol over 4 consecutive days. Then, 25,000 IU per week up to 6 weeks. vs. Placebo	63.24 ± 14.46	17.87 ± 10.15/NA	68.73 ± 10.97/NA	16.87 ± 9.48/NA	3/NA/5: 22 vs. 4/NA/2: 21
Annweiler C et al. (5), French	Cohort study, hospitalized patients	Oral 50,000 IU cholecalciferol per month, or 80,000 IU or 100,000 IU, or 200,000 IU every 2–3 months, or 800 IU daily. vs. Without vitamin D supplements	87.7 ± 5.4	24.64 ± 14.16/NA	88.6 ± 5.7	29.56 ± 12.84/NA	16/NA/NA:67 vs. 13/NA/NA: 28
Annweiler C et al. (6), French	Cohort Study, COVID-19 patients in the nursing home	Oral 80,000 IU cholecalciferol vs. Without vitamin D supplements	87.7 ± 9.3	NA	87.4 ± 7.2	NA	10/NA/NA: 57 vs. 5/NA/NA: 9
Annweiler G et al. (11), France	Cohort Study, Hospitalized patients	Oral 80,000 IU cholecalciferol within a few hours of the diagnosis vs. Without vitamin D supplements	85 (84–89)	NA	88 (84–92)	NA	3/NA/NA:45 vs. 10/NA/NA: 32
Güven et al. (12), Turkey	Cohort Study, Hospitalized patients	Inject 300,000 IU cholecalcifero in the first 24 h of admission vs. Without vitamin D supplements	74 (60–81)	6.65 (5.06–9.1)/NA	75 (62–83)	7.14 (5.17–8.21)/NA	43/44/NA:113 vs. 30/31/NA:62
Xavier et al. (13), Spain	Cohort Study, Hospitalized patients	Oral 532 μg calcifediol on day 1 plus 266 μg on days 3, 7, 15, and 30. vs. Without vitamin D supplements	61.81 ± 15.5	13(8–24)/NA	62.41 ± 17.2	12 (8–19)/NA	21/NA/20:447 vs. 47/NA/82: 391
Soliman et al. (25), Egypt	Cohort Study, Hospitalized patients with type 2 diabetes	Inject a single dose of 200,000 IU cholecalciferol vs. Placebo	71.30 ± 4.16	10.4 ± 1.3/20.54 ± 3.00	70.19 ± 4.57	21.17 ± 3.96/21.23 ± 3.98	7/14/NA: 40 vs. 3/7/NA: 16
Alcala-Diaz et al. (26), Spain	Cohort Study, Hospitalized patients	Oral 0.532 mg calcifediol at day 0, 0.266 mg on days 3 and 7, and then weekly. vs. Without vitamin D supplements.	69 ± 15	NA	67 ± 16	NA	4/3/NA: 79 vs. 90/26/NA: 458
Jevalikar et al. (27), India	Cohort Study, Hospitalized patients	A single oral dose of 60,000 IU cholecalciferol vs. Without vitamin D supplements.	45.5 ± 18.2	<20/NA	48.8 ± 14.7	<20/NA	1/NA/16:128 vs. 3/NA/13: 69

^a^Only 16 participants from two study sites had their blood samples drawn for measurement of serum 25(OH)D. Calcifediol, 25-hydroxyvitamin D3; calcitriol, 1,25-Dihydroxyvitamin D3; cholecalciferol, vitamin D3; IQR, interquartile range; NA, not available. This table presented data as mean ± SD, or median (IQR).

**TABLE 2:** The Grading of Recommendation, Assessment, Development, and Evaluation (GRADE).

Outcome	Certainty assessment							No. of patients		Effect	Certainty
	Study design	No. of studies	Risk of bias	Inconsistency	Indirectness	Imprecision	Other considerations	Vitamin D	Control	Relative risk (95% CI)	
Mortality	Cohort studies	8	Serious^a^	Not serious	Serious^b^	Not serious	None	105/976 (10.8%)	201/1065(18.9%)	RR 0.33(0.23–0.47)	⊕⊕○○ Low
	Randomized controlled trials	8	Serious^a^	Not serious	Serious^b^	Serious^d^	None	59/677 (8.7%)	65/641(10.1%)	RR 0.94(0.69–1.29)	⊕○○○ Very low
ICU admission	Cohort studies	2	Serious^a^	Seriousc^c^	Serious^b^	Serious^d^	None	36/575 (6.3%)	95/460 (20.7%)	RR 0.32(0.08–1.29)	⊕○○○ Very low
	Randomized controlled trials	6	Serious^a^	Serious^e^	Serious^b^	Serious^d^	None	86/605(14.2%)	103/562 (18.3%)	RR 0.64(0.38–1.08)	⊕○○○ Very low
Mechanical ventilation or intubation	Cohort studies	3	Serious^a^	Not serious	Serious^b^	Serious^d^	None	64/536 (11.9%)	61/232 (26.3%)	RR 0.93(0.55–1.58)	⊕○○○ Very low
	Randomized controlled trials	5	Serious^a^	Not serious	Serious^b^	Serious^d^	None	49/331(14.8%)	69/325 (21.2%)	RR 0.66(0.39–1.10)	⊕○○○ Very low

CI, confidence interval; RR, risk ratio.

^a^Some do not concern with the method of randomization used/allocation concealment/blinding of participants/blinding of outcome assessment/selective reporting.

^b^There were differences in vitamin D dosages and duration.

^c^*I*^2^ = 90%;

^d^The confidence interval was not narrow enough for us to be confident that this is the effect or does it reduce and has no effect.

^e^i^2^ = 60%. Grades of evidence: High: Further research is very unlikely to change our confidence in the estimate of effect; Moderate: Further research is likely to have an important impact on our confidence in the estimate of effect and may change the estimate; low: Further research is very likely to have an important impact on our confidence in the estimate of effect and is likely to change the estimate; Very low: Any estimate of effect is very uncertain. The number of plus symbols shows the degree of certainty, more plus symbols indicate a higher degree of certainty.

## Results

### Literature search

**FIGURE 1:**
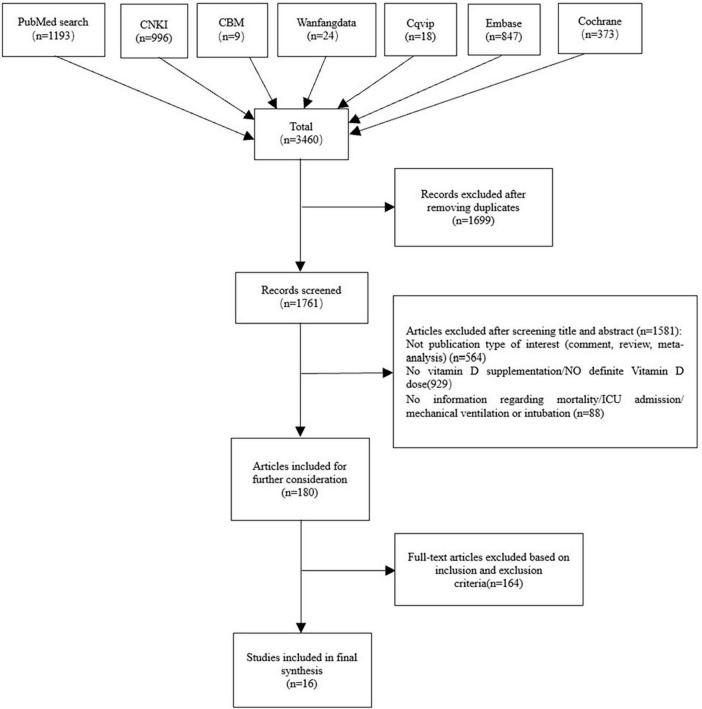
Flow chart of literature searching and screening.

A comprehensive literature search was conducted, resulting in the identification of 3,460 citations. Upon manual removal of 1,699 duplicates, screening the remaining titles and abstracts resulted in the selection of 180 articles. Further evaluation of full text resulted in the inclusion of 16 studies in the final analysis (Figure 1), consisting of 8 RCTs (8–10, 20–24), and 8 cohort studies (5, 6, 11–13, 25–27).

### Study characteristics and risk of bias of the included literature

Table 1 presents the characteristics of the included studies. The RCTs included 1,318 subjects, with 677 in the vitamin D supplementation group and 641 in the control group. The cohort studies included 2,041 subjects, with 976 in the vitamin D supplementation group and 1,065 in the control group. All the studies were carried out in hospitals, except for one which was conducted in a nursing home in France (6). The sample sizes of RCTs ranged from 43 to 543, with mean or median ages ranging from 10.7 to 69 years and follow-up from 7 days to 4 months (8–10, 20–24). Cholecalciferol was administered in the intervention arm of six RCTs (9, 10, 20–22, 24), while calcifediol (23) and calcitriol (8) were used in the remaining two RCTs. The sample sizes of the eight cohort studies ranged from 48 to 785, with mean ages ranging from 45.5 to 87.7 years, and follow-up from 5 days to 3 months. Cholecalciferol was administered in the intervention arm of six cohort studies (5, 6, 11, 13, 25, 27), and calcifediol was administered in the remaining two studies (12, 26). Out of the 16 included studies, only 10 reported the mean baseline levels of serum 25(OH)D, which ranged from 6.65 to 32.5 ng/ml in the intervention groups and 7.14 to 30.5 ng/ml in the control groups (Table 1).

Four RCTs had a low risk of bias (10, 20, 22, 24), one was at a high risk of bias (21) and the rest three studies had an uncertain risk of bias (8, 9, 23) (Supplementary Figures 1, 2). Six cohort studies had a moderate risk of bias (5, 12, 13, 25–27), and the other two had a serious risk of bias (6, 11) (Supplementary Figure 3).

### GRADE assessment

The quality of evidence was assessed using the GRADE methods, as presented in Table 2. The certainty of the evidence for mortality (RCTs were very low, cohort studies were low), ICU admission (both RCTs and cohort studies were very low), and mechanical ventilation or intubation (both RCTs and cohort studies were very low) were rated as low to very low due to the heterogeneity in drug type and dosing, population characteristic, and the quality of the included studies.

## Outcomes of meta-analyses

### Effect of vitamin D supplementation on mortality

**FIGURE 2:**
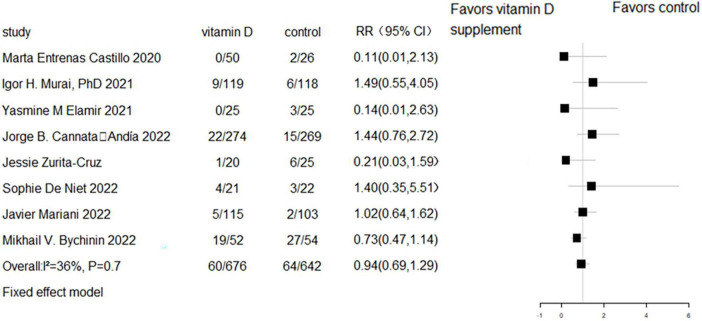
Forest plot of RCTs for vitamin D supplementation on mortality.

**FIGURE 3:**
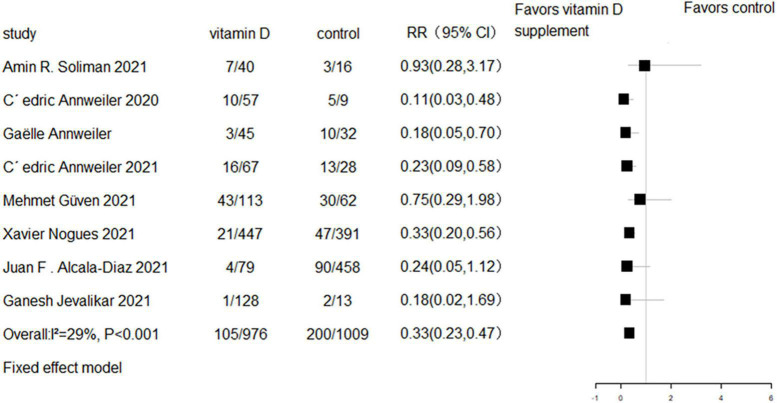
Forest plot of cohort studies for vitamin D supplementation on mortality (All cohort studies).

**FIGURE 4:**
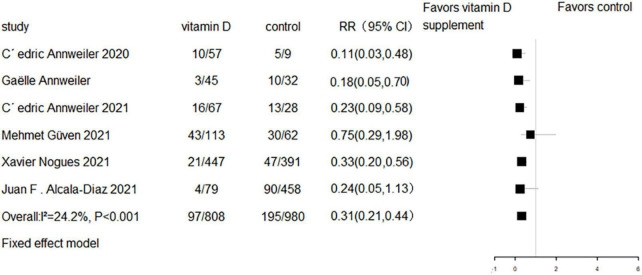
Forest plot of cohort studies for vitamin D supplementation on mortality (studies with adjusted RR values only).

All eight RCTs (*n* = 1,318) and eight cohort studies (*n* = 2,041) reported the effect of vitamin D supplementation on mortality in COVID-19 patients. The meta-analysis of RCTs indicated no significant difference in mortality between the intervention group and control group (RR = 0.94, 95% CI 0.69–1.29, *P* = 0.7; fixed effect model; very low-certainty evidence; Figure 2). For the eight cohort studies, three reported adjusted HRs, another three reported adjusted ORs, and the remaining two studies reported the number of deaths. Subjects with vitamin D supplementation had significantly lower mortality than the control group (RR = 0.33, 95% CI 0.23–0.47, *P* < 0.001; fixed effect model; low-certainty evidence; Figure 3). The results remained consistent even after excluding studies that reported unadjusted RRs or numbers of deaths (RR = 0.31, 95% CI 0.21–0.44, *P* < 0.001; fixed effect model; Figure 4).

**FIGURE 5:**
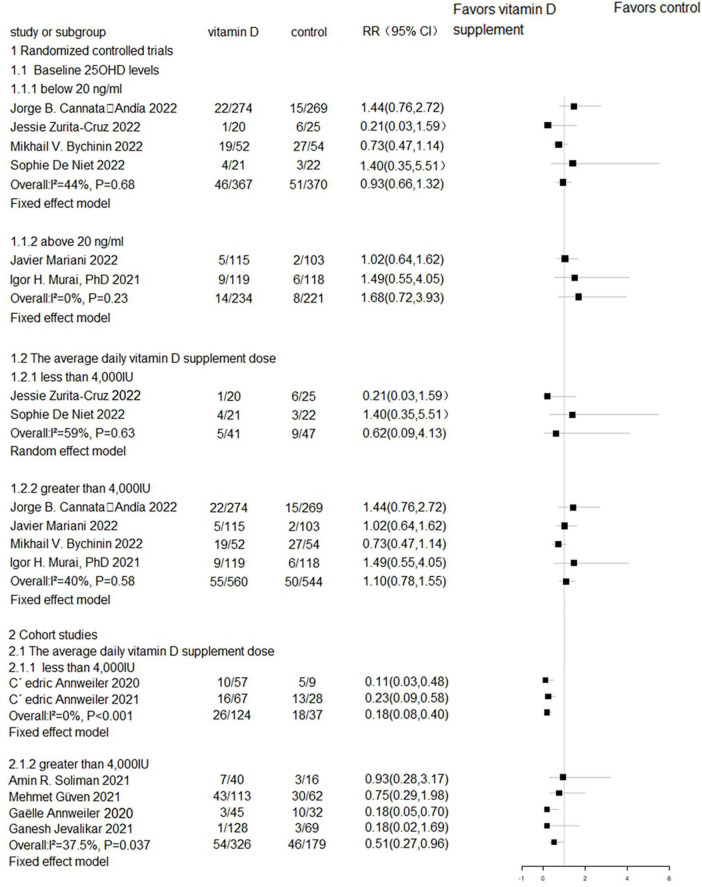
Subgroup analyses of mortality.

We performed subgroup analyses to investigate the association between the average daily vitamin D supplement dose and serum 25(OH)D levels with mortality. The results revealed no significant differences in mortality between individuals with baseline 25OHD levels below 20 ng/ml (RR = 0.93, 95% CI 0.66–1.32, *P* = 0.68) (9, 21, 22, 24) and those with levels above 20 ng/ml (RR = 1.68, 95% CI 0.72–3.93, *P* = 0.23) (10, 20), or between individuals receiving average daily vitamin D supplementation doses less than 4,000 IU (21, 24) (RR = 0.62, 95% CI 0.09–4.13, *P* = 0.63) and those receiving doses greater than 4,000 IU (9, 10, 20, 22) (RR = 1.10, 95% CI 0.78–1.55, *P* = 0.58). However, the results from cohort studies indicated that there was a significant reduction in mortality among individuals receiving average daily vitamin D supplementation doses less than 4,000 IU (5, 6) (RR = 0.18, 95% CI 0.08–0.40, *P* < 0.001) and those receiving doses greater than 4,000 IU (11, 12, 25, 27) (RR = 0.51, 95% CI 0.27–0.96, *P* = 0.037) (Figure 5).

### The effect of vitamin D supplementation on ICU admission

**FIGURE 6:**
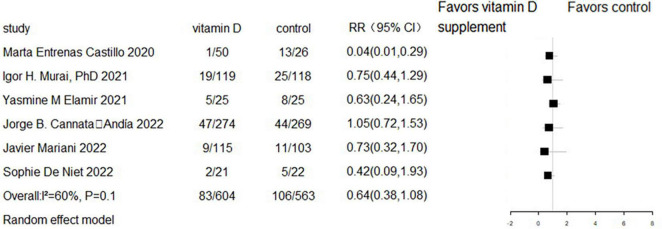
Forest plot of RCTs for vitamin D supplementation on ICU admission.

**FIGURE 7:**
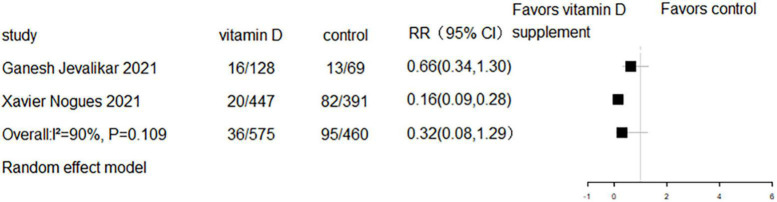
Forest plot of cohort studies for vitamin D supplementation on ICU admission.

Six RCTs and two cohort studies reported the effect of vitamin D supplementation on ICU admission. Meta-analyses showed that there was no difference in ICU admission between the vitamin D supplementation and control groups in either RCTs (RR = 0.64, 95%CI 0.38–1.08, *P* = 0.10; random effect model; very low-certainty evidence; Figure 6) or cohort studies (RR = 0.32, 95% CI 0.08–1.29, *P* = 0.109; random effect model; very low-certainty evidence; Figure 7).

### The effect of vitamin D supplementation on mechanical ventilation or intubation

**FIGURE 8:**
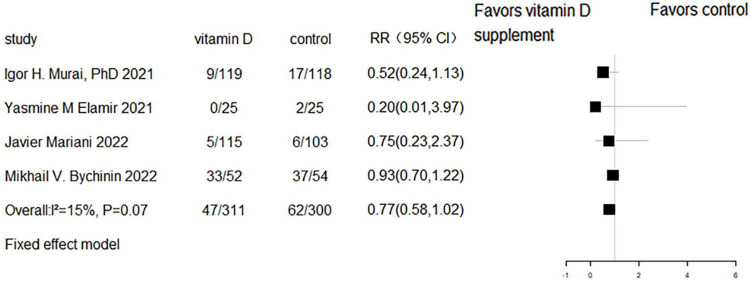
Forest plot of RCTs for vitamin D supplementation on mechanical ventilation or intubation.

**FIGURE 9:**
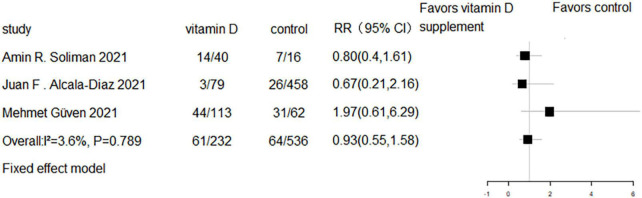
Forest plot of cohort studies for vitamin D supplementation on mechanical ventilation or intubation.

Five RCTs and three cohort studies reported the effect of vitamin D supplementation on mechanical ventilation or intubation. Meta-analyses of RCTs (RR = 0.77, 95% CI 0.58–1.02, *P* = 0.07; fixed effect model; very low-certainty evidence; Figure 8) and cohorts (RR = 0.93, 95% CI 0.55–1.58, *P* = 0.789; fixed effect model; very low-certainty evidence; Figure 9) showed that there was no difference in mechanical ventilation or intubation rate in COVID-19 patients with or without vitamin D supplementation.

### Publication bias

**FIGURE 10:**
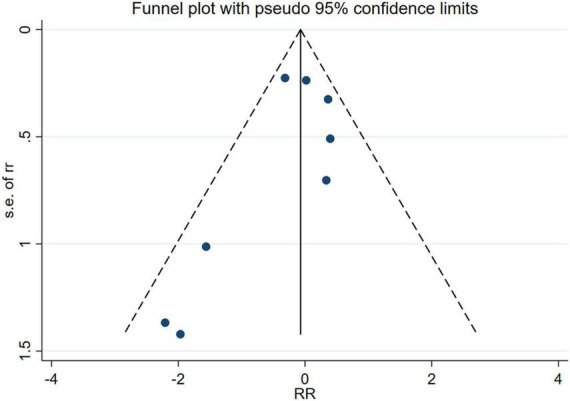
Funnel plot of RCTs.

**FIGURE 11:**
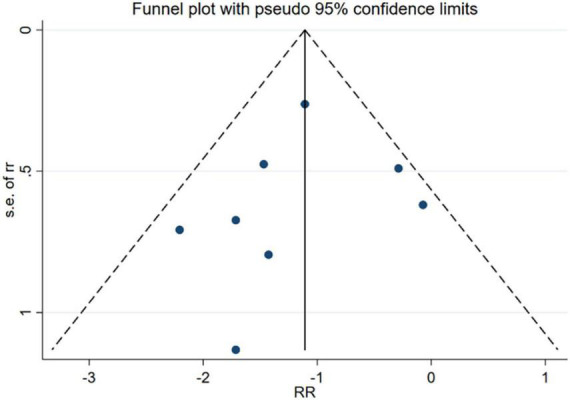
Funnel plot of cohort studies.

No evidence of publication bias was identified through the analysis of the funnel plots (RCTs’ Egger’s test *P* = 0.266, Figure 10; cohort’s Egger’s test *P* = 0.604, Figure 11).

## Discussion

This present meta-analysis included eight RCTs (8–10, 20–24) and eight cohort studies (5, 6, 11–13, 25–27) involving a total of 3,359 subjects. The results of pooled data indicated that vitamin D supplementation did not significantly reduce mortality, ICU admission, or rates of mechanical ventilation and intubation in COVID-19 patients. The conclusion should be interpreted with caution due to the low quality of the studies included, their small sample sizes, and significant baseline heterogeneity in baseline factors, including drug type and dosing, and population characteristics.

It is widely recognized that vitamin D can regulate the immune system, and its deficiency has been linked to an increased risk of developing the “cytokine storm” associated with COVID-19 (28). Recent reviews of the literature have also suggested that optimizing vitamin D levels in the general population may have served as a protective measure against COVID-19 infection (29, 30). Our study is not the first meta-analysis of vitamin D supplementation in COVID-19 patients. A previous meta-analysis published in 2021 (31) comprising 3 RCTs (20, 23, 32) and 2 cohort studies (6, 11) found that vitamin D supplementation did not result in a significant reduction in mortality, ICU admission rates, or mechanical ventilation (31). Another meta-analysis published in 2021 (33) involving 2 RCTs (20, 23) and 1 case-control study (34) showed that vitamin D supplementation resulted in comparable mortality but lower intensive care unit needs in patients with COVID-19. These two meta-analyses pooled studies with different study types and had much smaller sample sizes than our study. Our meta-analysis was based on a comprehensive search strategy and use established scales to assess the quality of research and strength of evidence. Furthermore, adjusted ORs were used to minimize bias in cohort studies. As a result, our conclusions are more robust and reliable compared to previous meta-analyses.

The pooled analysis found an inconsistent effect of vitamin D supplementation on mortality in cohort studies and RCTs. Although evidence showed that patients receiving higher cumulative doses and average daily doses had a greater decrease in COVID-19 infection rates compared to those receiving lower doses (35), subgroup analysis indicated that there were no significant differences in mortality between individuals with lower or higher baseline 25OHD levels, as well as those receiving small or larger vitamin D supplementation doses in RCTs. Nevertheless, the results from RCTs were more reliable due to the superior methodology.

There are some limitations in this meta-analysis, including the small sample sizes and low quality of the included RCTs and cohort studies, as well as the lack of complete information regarding the study population, such as race, sex, and 25(OH)D level before and after vitamin D supplementation. There was also significant heterogeneity among the included studies in terms of drug type and dosing, population features, and COVID-19 severity and treatment strategies.

In conclusion, while the results of this meta-analysis suggest that vitamin D supplementation may not significantly reduce mortality, ICU admission, and rates of mechanical ventilation intubation in COVID-19 patients, additional well-designed RCTs with large sample sizes are needed to further explore the potential benefit of vitamin D supplementation in this population.

## Data availability statement

The original contributions presented in this study are included in the article/Supplementary material, further inquiries can be directed to the corresponding author.

## Author contributions

YZ, JL, and QW designed the review. YZ and JL conducted the systematic review and extracted data. MY and YZ performed the data analysis. JL and QW wrote the manuscript. QW had primary responsibility for final content. All authors read and approved the final manuscript.
