# Supplementary material for: Effect of vitamin D supplementation on COVID-19 patients: A systematic review and meta-analysis
Source: Front Nutr. 2023 Mar 7;10:1131103. doi: 10.3389/fnut.2023.1131103 (PMC10027755; doi:10.3389/fnut.2023.1131103)
Supplement: Supplementary file 1 [file Data_Sheet_1.docx]

**Table of contents:**

Figure S1: Risk of bias summary based on Cochrane Systematic Review Guidelines for each included study (green for low risk of bias,yellow for unclear risk of bias and red for high risk of bias) included in this review.

Figure S2: Risk of bias graph review authors judgements about each risk of bias item presented as percentages across various study designs.

Figure S3: A summary plot and B Traffic light plot highlighting quality of studies using Robin-I tool.

Text S1:Full electronic search strategy.


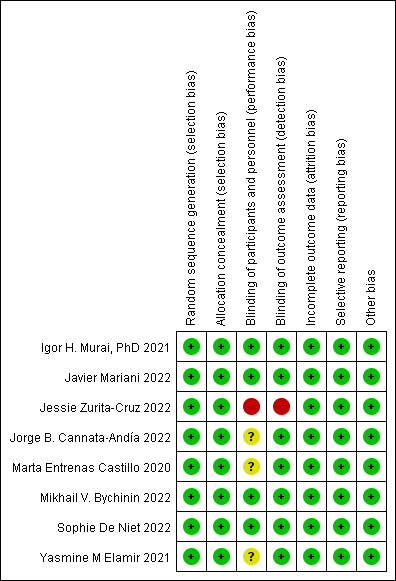


Figure S1: Risk of bias summary based on Cochrane Systematic Review Guidelines for each included study (green for low risk of bias,yellow for unclear risk of bias and red for high risk of bias) included in this review.


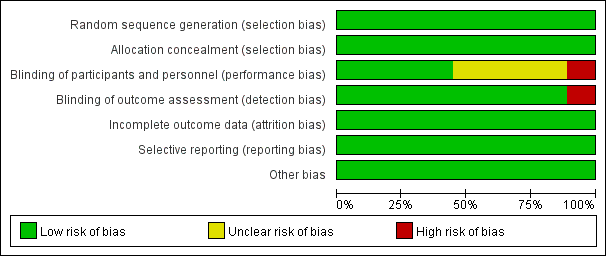


Figure S2: Risk of bias graph review authors judgements about each risk of bias item presented as percentages across various study designs.

**A**


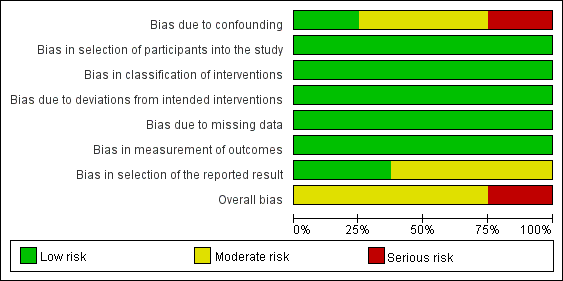


**B**


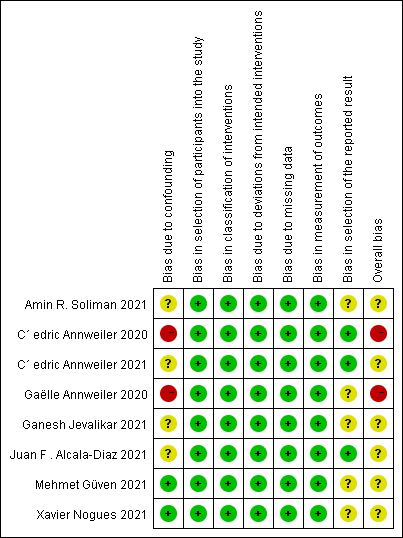


Figure S3: A summary plot and B Traffic light plot highlighting quality of studies using Robin-I tool.

**Text S1:Full electronic search strategy**

CNKI

( ( ( ( ( ( ( ( ( ( ( ( 主题%='维生素D' or 题名%='维生素D' or title=xls('维生素D') or v_subject=xls('维生素D') ) OR ( 主题%='胆钙化醇' or 题名%='胆钙化醇' or title=xls('胆钙化醇') or v_subject=xls('胆钙化醇') ) ) OR ( 主题%='麦角钙化醇' or 题名%='麦角钙化醇' or title=xls('麦角钙化醇') or v_subject=xls('麦角钙化醇') ) ) OR ( 主题%='羟胆钙化醇' or 题名%='羟胆钙化醇' or title=xls('羟胆钙化醇') or v_subject=xls('羟胆钙化醇') ) ) OR ( 主题%='骨化二醇' or 题名%='骨化二醇' or title=xls('骨化二醇') or v_subject=xls('骨化二醇') ) ) OR ( 主题%='25羟维生素D' or 题名%='25羟维生素D' or title=xls('25羟维生素D') or v_subject=xls('25羟维生素D') ) ) OR ( 主题%='1,25二羟维生素D' or 题名%='1,25二羟维生素D' or title=xls('1,25二羟维生素D') or v_subject=xls('1,25二羟维生素D') ) ) OR ( 主题%='骨化三醇' or 题名%='骨化三醇' or title=xls('骨化三醇') or v_subject=xls('骨化三醇') ) ) OR ( 主题%='阿法骨化醇' or 题名%='阿法骨化醇' or title=xls('阿法骨化醇') or v_subject=xls('阿法骨化醇') ) ) OR ( 主题%='帕立骨化醇' or 题名%='帕立骨化醇' or title=xls('帕立骨化醇') or v_subject=xls('帕立骨化醇') ) ) AND ( ( ( ( 主题%='新冠肺炎' or 题名%='新冠肺炎' or title=xls('新冠肺炎') or v_subject=xls('新冠肺炎') ) OR ( 主题%='新型冠状病毒肺炎' or 题名%='新型冠状病毒肺炎' or title=xls('新型冠状病毒肺炎') or v_subject=xls('新型冠状病毒肺炎') ) ) OR ( 主题%='新型冠状病毒' or 题名%='新型冠状病毒' or title=xls('新型冠状病毒') or v_subject=xls('新型冠状病毒') ) ) OR ( 主题%=xls('COVID-19') or 题名%=xls('COVID-19') or title='COVID-19' or v_subject='COVID-19' ) ) )

WanFang Data

(主题:(维生素D or 胆钙化醇 or 麦角钙化醇 or 羟胆钙化醇 or 骨化二醇 or 25羟维生素D or 1,25二羟维生素D or 骨化三醇 or 阿法骨化醇 or 帕立骨化醇) and 主题:(新冠肺炎 or 新型冠状病毒肺炎 or 新型冠状病毒 or COVID-19)

Cqvip

题名或关键词=维生素D+胆钙化醇+麦角钙化醇+羟胆钙化醇+骨化二醇+25羟维生素D+1,25二羟维生素D+骨化三醇+阿法骨化醇+帕立骨化醇 AND 题名或关键词=新冠肺炎+新型冠状病毒肺炎+新型冠状病毒+COVID-19

CBM

(("新型冠状病毒肺炎"[常用字段:智能]) OR ("新型冠状病毒"[常用字段:智能]) OR ("COVID-19"[常用字段:智能]) OR ("新冠肺炎"[常用字段:智能])) AND (("帕立骨化醇"[常用字段:智能]) OR ("阿法骨化醇"[常用字段:智能]) OR ("骨化三醇"[常用字段:智能]) OR ("1,25二羟维生素D"[常用字段:智能]) OR ("25羟维生素D"[常用字段:智能]) OR ("骨化二醇"[常用字段:智能]) OR ("羟胆钙化醇"[常用字段:智能]) OR ("麦角钙化醇"[常用字段:智能]) OR ("胆钙化醇"[常用字段:智能]) OR ("维生素D"[不加权:扩展]))

Pubmed

((((((((((((((((((((((((((((((((((((COVID 19 Virus Disease[Title/Abstract]) OR (COVID 19[Title/Abstract])) OR (COVID-19 Virus Disease[Title/Abstract])) OR (COVID-19 Virus Diseases[Title/Abstract])) OR (Disease, COVID-19 Virus[Title/Abstract])) OR (Virus Disease, COVID-19[Title/Abstract])) OR (COVID-19 Virus Infection[Title/Abstract])) OR (COVID 19 Virus Infection[Title/Abstract])) OR (COVID-19 Virus Infections[Title/Abstract])) OR (Infection, COVID-19 Virus[Title/Abstract])) OR (Virus Infection, COVID-19[Title/Abstract])) OR (2019-nCoV Infection[Title/Abstract])) OR (2019 nCoV Infection[Title/Abstract])) OR (2019-nCoV Infections[Title/Abstract])) OR (Infection, 2019-nCoV[Title/Abstract])) OR (Coronavirus Disease-19[Title/Abstract])) OR (Coronavirus Disease 19[Title/Abstract])) OR (2019 Novel Coronavirus Disease[Title/Abstract])) OR (2019 Novel Coronavirus Infection[Title/Abstract])) OR (2019-nCoV Disease[Title/Abstract])) OR (2019 nCoV Disease[Title/Abstract])) OR (2019-nCoV Diseases[Title/Abstract])) OR (Disease, 2019-nCoV[Title/Abstract])) OR (COVID19[Title/Abstract])) OR (Coronavirus Disease 2019[Title/Abstract])) OR (Disease 2019, Coronavirus[Title/Abstract])) OR (SARS Coronavirus 2 Infection[Title/Abstract])) OR (SARS-CoV-2 Infection[Title/Abstract])) OR (Infection, SARS-CoV-2[Title/Abstract])) OR (SARS CoV 2 Infection[Title/Abstract])) OR (SARS-CoV-2 Infections[Title/Abstract])) OR (COVID-19 Pandemic[Title/Abstract])) OR (COVID 19 Pandemic[Title/Abstract])) OR (COVID-19 Pandemics[Title/Abstract])) OR (Pandemic, COVID-19[Title/Abstract]))) AND ((((((((((((((((((((((((vitamin D[Title/Abstract]) OR (Vitamin D 3[Title/Abstract])) OR (Vitamin D3[Title/Abstract])) OR (Vitamin D2[Title/Abstract])) (cholecalciferol[Title/Abstract])) OR (ergocalciferol[Title/Abstract])) OR (hydroxycholecalciferol[Title/Abstract])) OR (calcifediol[Title/Abstract])) OR (calcidiol[Title/Abstract])) OR (25-hydroxyvitamin D[Title/Abstract])) OR (25-Hydroxyvitamin D3[Title/Abstract])) OR (25Hydroxyvitamin D 3[Title/Abstract])) OR (1, 25-dihydroxyvitamin D[Title/Abstract])) OR (1,25-Dihydroxyvitamin[Title/Abstract])) OR (1,25-Dihydroxy-cholecalciferol[Title/Abstract])) OR (1alpha,25-Dihydroxycholecalciferol[Title/Abstract])) OR (1 alpha,25-Dihydroxyvitamin[Title/Abstract])) OR (Dihydroxycholecalciferol[Title/Abstract])) OR (Dihydroxyvitamins D[Title/Abstract])) OR (Dihydroxyvitamin[Title/Abstract])) OR (calcitriol[Title/Abstract])) OR (alfacalcidol[Title/Abstract])) OR (24,25-Dihydroxyvitamin D 3[Title/Abstract])) OR (24,25-Dihydroxyvitamin[Title/Abstract])) OR (paricalcitol[Title/Abstract]))))

Embase

| #3 | #1 AND #2 |
| --- | --- |
| #2 | 'covid 19 virus disease':ab,ti OR 'covid 19':ab,ti OR 'covid-19 virus disease':ab,ti OR 'covid-19 virus diseases':ab,ti OR 'disease, covid-19 virus':ab,ti OR 'virus disease, covid-19':ab,ti OR 'covid-19 virus infection':ab,ti OR 'covid 19 virus infection':ab,ti OR 'covid-19 virus infections':ab,ti OR 'infection, covid-19 virus':ab,ti OR 'virus infection, covid-19':ab,ti OR '2019-ncov infection':ab,ti OR '2019 ncov infection':ab,ti OR '2019-ncov infections':ab,ti OR 'infection, 2019-ncov':ab,ti OR 'coronavirus disease-19':ab,ti OR 'coronavirus disease 19':ab,ti OR '2019 novel coronavirus disease':ab,ti OR '2019 novel coronavirus infection':ab,ti OR '2019-ncov disease':ab,ti OR '2019 ncov disease':ab,ti OR '2019-ncov diseases':ab,ti OR 'disease, 2019-ncov':ab,ti OR covid19:ab,ti OR 'coronavirus disease 2019':ab,ti OR 'disease 2019, coronavirus':ab,ti OR 'sars coronavirus 2 infection':ab,ti OR 'sars-cov-2 infection':ab,ti OR 'infection, sars-cov-2':ab,ti OR 'sars cov 2 infection':ab,ti OR 'sars-cov-2 infections':ab,ti OR 'covid-19 pandemic':ab,ti OR 'covid 19 pandemic':ab,ti OR 'covid-19 pandemics':ab,ti OR 'pandemic, covid-19':ab,ti |
| #1 | 'vitamin d':ab,ti OR 'vitamin d 3':ab,ti OR 'vitamin d 2':ab,ti OR 'vitamin d3':ab,ti OR cholecalciferol:ab,ti OR ergocalciferol:ab,ti OR hydroxycholecalciferol:ab,ti OR calcifediol:ab,ti OR calcidiol:ab,ti OR '25-hydroxyvitamin d':ab,ti OR '25-hydroxyvitamin d3':ab,ti OR '25hydroxyvitamin d 3':ab,ti OR '1, 25-dihydroxyvitamin d':ab,ti OR '1,25 dihydroxyvitamin':ab,ti OR '1,25 dihydroxy cholecalciferol':ab,ti OR '1alpha,25 dihydroxycholecalciferol':ab,ti OR '1 alpha,25-dihydroxyvitamin':ab,ti OR dihydroxycholecalciferol:ab,ti OR 'dihydroxyvitamins d':ab,ti OR dihydroxyvitamin:ab,ti OR calcitriol:ab,ti OR alfacalcidol:ab,ti OR '24,25-dihydroxyvitamin d 3':ab,ti OR '24,25 dihydroxyvitamin':ab,ti OR paricalcitol:ab,ti |

Cochrane

#1 (vitamin D):ti,ab,kw OR (Vitamin D 3):ti,ab,kw OR (Vitamin D3):ti,ab,kw OR (Vitamin D2):ti,ab,kw OR (Cholecalciferol):ti,ab,kw OR (Hydroxycholecalciferol):ti,ab,kw OR (Ergocalciferol):ti,ab,kw OR (Calcifediol):ti,ab,kw OR (Calcidiol):ti,ab,kw OR ("25-hydroxyvitamin D"):ti,ab,kw OR ("25-Hydroxyvitamin D3"):ti,ab,kw OR ("25Hydroxyvitamin D 3"):ti,ab,kw OR ("1, 25-dihydroxyvitamin D"):ti,ab,kw OR ("1,25-Dihydroxyvitamin"):ti,ab,kw OR ("1,25-Dihydroxy-cholecalciferol"):ti,ab,kw OR ("1alpha,25-Dihydroxycholecalciferol"):ti,ab,kw OR ("1 alpha,25-Dihydroxyvitamin"):ti,ab,kw OR (Dihydroxyvitamins D):ti,ab,kw OR (Dihydroxycholecalciferol):ti,ab,kw OR (Dihydroxyvitamin):ti,ab,kw OR (Calcitriol):ti,ab,kw OR (Alfacalcidol):ti,ab,kw OR ("24,25-Dihydroxyvitamin D 3"):ti,ab,kw OR ("24,25-Dihydroxyvitamin"):ti,ab,kw OR (paricalcitol):ti,ab,kw 17843

#2 ("COVID 19 Virus Disease"):ti,ab,kw OR ("COVID 19"):ti,ab,kw OR ("COVID-19 Virus Disease"):ti,ab,kw OR ("COVID-19 Virus Diseases"):ti,ab,kw OR ("Disease, COVID-19 Virus"):ti,ab,kw OR ("Virus Disease, COVID-19"):ti,ab,kw OR ("COVID-19 Virus Infection"):ti,ab,kw OR ("COVID 19 Virus Infection"):ti,ab,kw OR ("COVID-19 Virus Infections"):ti,ab,kw OR ("Infection, COVID-19 Virus"):ti,ab,kw OR ("Virus Infection, COVID-19"):ti,ab,kw OR ("2019-nCoV Infection"):ti,ab,kw OR ("2019 nCoV Infection"):ti,ab,kw OR ("2019-nCoV Infections"):ti,ab,kw OR ("Infection, 2019-nCoV"):ti,ab,kw OR ("Coronavirus Disease-19"):ti,ab,kw OR ("Coronavirus Disease 19"):ti,ab,kw OR ("2019 Novel Coronavirus Disease"):ti,ab,kw OR ("2019 Novel Coronavirus Infection"):ti,ab,kw OR ("2019-nCoV Disease"):ti,ab,kw OR ("2019 nCoV Disease"):ti,ab,kw OR ("2019-nCoV Diseases"):ti,ab,kw OR ("Disease, 2019-nCoV"):ti,ab,kw OR ("COVID19"):ti,ab,kw OR ("Coronavirus Disease 2019"):ti,ab,kw OR ("Disease 2019, Coronavirus"):ti,ab,kw OR ("SARS Coronavirus 2 Infection"):ti,ab,kw OR ("SARS-CoV-2 Infection"):ti,ab,kw OR ("Infection, SARS-CoV-2"):ti,ab,kw OR ("SARS CoV 2 Infection"):ti,ab,kw OR ("SARS-CoV-2 Infections"):ti,ab,kw OR ("COVID-19 Pandemic"):ti,ab,kw OR ("COVID 19 Pandemic"):ti,ab,kw OR ("COVID-19 Pandemics"):ti,ab,kw OR ("Pandemic, COVID-19"):ti,ab,kw 10120

#3 #1 AND #2 170
